# Type I toxin-dependent generation of superoxide affects the persister life cycle of *Escherichia coli*

**DOI:** 10.1038/s41598-019-50668-1

**Published:** 2019-10-03

**Authors:** Daniel Edelmann, Bork A. Berghoff

**Affiliations:** Institute for Microbiology and Molecular Biology, Justus Liebig University Giessen, 35392 Giessen, Germany

**Keywords:** Bacterial physiology, Antibiotics

## Abstract

Induction of growth stasis by bacterial toxins from chromosomal toxin-antitoxin systems is suspected to favor formation of multidrug-tolerant cells, named persisters. Recurrent infections are often attributed to resuscitation and regrowth of persisters upon termination of antibiotic therapy. Several lines of evidence point to oxidative stress as a crucial factor during the persister life cycle. Here, we demonstrate that the membrane-depolarizing type I toxins TisB, DinQ, and HokB have the potential to provoke reactive oxygen species formation in *Escherichia coli*. More detailed work with TisB revealed that mainly superoxide is formed, leading to activation of the SoxRS regulon. Deletion of the genes encoding the cytoplasmic superoxide dismutases SodA and SodB caused both a decline in TisB-dependent persisters and a delay in persister recovery upon termination of antibiotic treatment. We hypothesize that expression of depolarizing toxins during the persister formation process inflicts an oxidative challenge. The ability to counteract oxidative stress might determine whether cells will survive and how much time they need to recover from dormancy.

## Introduction

Multidrug-tolerant persister cells were found in every bacterial population examined so far. Their generation may be seen as a bet-hedging strategy to maintain survival on the population level in unpredictable environments^1–4^. Even though persister cells might be very diverse in terms of physiology, some general features have emerged: (i) persisters are phenotypic variants that are genetically identical to their non-persistent siblings, (ii) a reduced growth rate favors the persister state, (iii) they are tolerant towards antibiotics and other cues, (iv) they are able to resume growth after the stress has ceased. Especially the latter feature sets them apart from ‘viable but non-culturable’ (VBNC) cells, that need specialized environmental conditions for resuscitation and re-growth^5^. The first persister gene, discovered in the early 1980s, was *hipA* in *Escherichia coli*^6^. HipA is the toxin moiety of the chromosomal toxin-antitoxin (TA) system HipAB. Even though not every chromosomal TA system is necessarily involved in the persister formation process, individual TA systems have been linked to bacterial persistence^7,8^.

TA systems are classified according to the nature of the antitoxin (RNA or protein) and the mechanism by which it controls its cognate toxin^9^. In type I TA systems, the antitoxin is an RNA that inhibits translation of the toxin mRNA to avoid toxin production under normal growth conditions. Toxin genes are often stress-inducible and elevated mRNA levels are only observed upon unfavorable conditions. Increasing toxin mRNA levels might at some point overcome the inhibitory action of the RNA antitoxin, ultimately leading to toxin production^10,11^. Toxins from type I TA systems are mostly small hydrophobic proteins (<50 amino acids) that target the inner membrane. In *E*. *coli*, for instance, this applies to TisB, DinQ, and HokB^12–14^. While transcription of *tisB* and *dinQ* is induced upon DNA damage as part of the SOS response^15,16^, *hokB* transcription depends on the GTPase ObgE and the alarmone (p)ppGpp^2^. All three toxins have the potential to disrupt the proton motive force (PMF), resulting in depolarization of the inner membrane and ATP depletion^2,12,14,17,18^. For HokB, it was even shown that mature pores are of a size that is compatible with ATP leakage^13^. Intracellular depletion of ATP triggers formation of multidrug-tolerant persister cells^19,20^, which also for TisB and HokB has been suggested to link toxin action to persistence^2,13,18,21^. However, a reduction in persister levels by deletion of the toxin gene was so far only demonstrated for TisB upon treatment with DNA-damaging antibiotics^18,21^, and remains to be tested for HokB and DinQ.

In aerobic environments, bacteria are exposed to reactive oxygen species (ROS), such as hydrogen peroxide, superoxide, and hydroxyl radicals. ROS are generally produced as a byproduct of aerobic metabolism by electron transfer to molecular oxygen within cells. Naturally occurring electron donors are metal centers, flavins and respiratory quinones^22^. Aerobic bacteria have, therefore, evolved mechanisms to counteract ROS and elicit specific oxidative stress responses to avoid extensive damage of macromolecules. As a first line of defense, ROS can be directly detoxified by specialized enzymes (e.g., superoxide dismutases, catalases, and peroxidases). Furthermore, bacteria exploit redox-balancing proteins (e.g., thioredoxins and glutaredoxins) and manifold enzymes involved in repair of damaged macromolecules to maintain survival. Endogenous production of ROS is often further enhanced by stressors that are – at first glance – unrelated to oxidative stress^23^. This applies to, e.g., antibiotics^24,25^, although the generation of ROS by bactericidal antibiotics is subject to scientific controversy^26–28^. However, in general it is expected that disturbance of metabolic pathways primes ROS production^29^. For instance, overexpression of several type I toxins in *Escherichia coli* caused increased mRNA levels of the oxidative stress regulator SoxS^30^. Transcription of *soxS* is induced by SoxR, a transcriptional regulator which is activated by redox-cycling drugs and superoxide^31,32^. It remains, therefore, an outstanding question whether type I toxins have the potential to trigger ROS formation and how elevated ROS levels affect persister formation and recovery from the persister state.

## Results

### Generation of reactive oxygen species coincides with membrane depolarization

**Figure 1:**
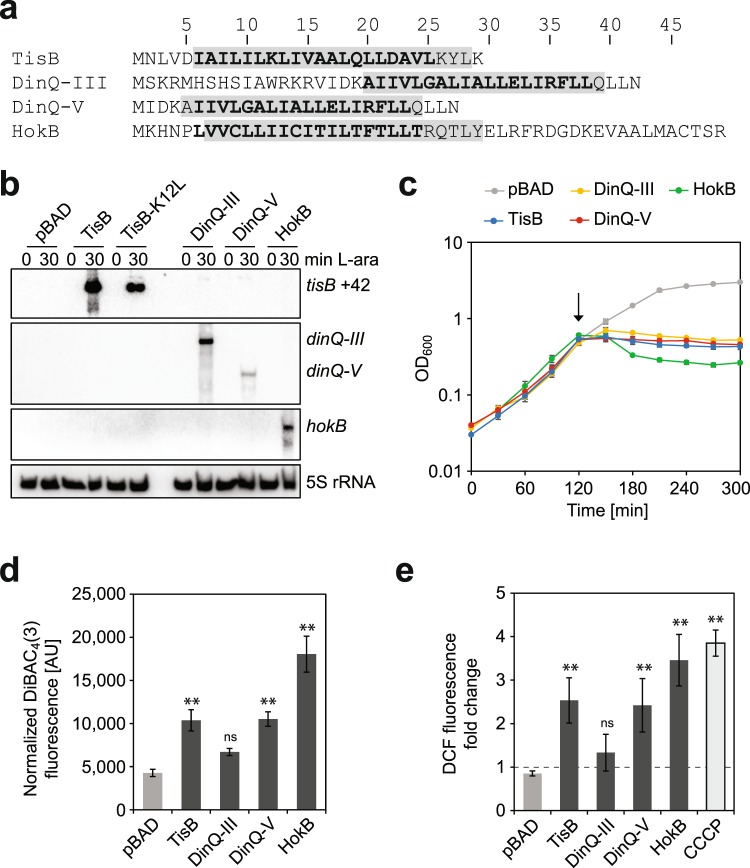
Depolarizing toxins provoke ROS formation. **(a)** Amino acid sequence of toxins selected for this study. Secondary structure predictions by Protter^59^ (bold characters) and TMHMM^60^ (light gray background) suggest single transmembrane helices for all toxins. **(b)** Nothern blot analysis of toxin mRNAs. Total RNA was isolated at 0 min and 30 min post induction with L-arabinose (0.2%). Analysis with toxin-specific probes confirmed specific toxin overexpression. An empty vector (pBAD) was used as negative control. 5 S rRNA was probed as loading control. Full scans of the Northern membranes can be found in Supplementary Fig. S6. **(c)** Growth curves upon overexpression of toxins. Toxin expression was induced in mid-exponential growth phase using 0.2% L-arabinose at 120-min time point (arrow). Growth was recorded by monitoring OD_600_ in 30-min time intervals. Data points represent the mean and error bars depict the standard deviation (n = 3). **(d)** Depolarization measurements upon overexpression of toxins. Toxins were overexpressed in mid-exponential phase for 60 min. Cell samples were stained with the fluorescent probe DiBAC_4_(3) and measured in a microplate reader. Fluorescence signals were OD_600_-normalized. Data represents the mean and error bars depict the standard deviation (n = 3). For statistical analysis one-way ANOVA with post-hoc Tukey HSD was performed. Significance levels for toxin samples vs. pBAD control are indicated (ns: not significant, **P < 0.01). **(e)** ROS measurements upon overexpression of toxins. Toxins were overexpressed in mid-exponential phase for 60 min. CCCP was applied for 30 min to provoke chemical depolarization of mid-exponential cultures. Pre- and post-treatment samples were stained with H_2_DCFDA. DCF fluorescence signals were measured in a microplate reader and OD_600_-normalized to calculate fold changes. Data represents the mean and error bars depict the standard deviation (n = 3). For statistical analysis one-way ANOVA with post-hoc Tukey HSD was performed. Significance levels for treatment samples vs. pBAD control are indicated (ns: not significant, **P < 0.01).

To test whether depolarizing type I toxins trigger ROS formation, pBAD plasmids with respective toxin genes under control of the P_BAD_ promoter were used for overexpression in *E*. *coli* K-12 wild type strain MG1655. Toxins TisB, HokB, and two DinQ variants with varying toxicity (less toxic DinQ-III and fully toxic DinQ-V)^14^ were selected. All four toxins contain a transmembrane helix (Fig. 1a) and are targeted towards the inner membrane^12–14^. Addition of the inducer L-arabinose caused specific transcription of toxin mRNAs (Fig. 1b), and resulted in the expected growth inhibition due to toxin production (Fig. 1c). HokB overexpression resulted in a drop in optical density, maybe due to leakage of cellular material through larger HokB pores^13^. The final optical density (300 min) was significantly lower than for all other toxins (*P* < 0.01, one-way ANOVA with post-hoc Tukey HSD). The potential-sensitive probe bis-(1,3-dibutylbarbituric acid) trimethine oxonol [DiBAC_4_(3)] was applied to monitor depolarization. Since DiBAC_4_(3) only enters depolarized cells, increasing cellular fluorescence is a direct measure for depolarization. All toxins, except DinQ-III, caused a significant increase in DiBAC_4_(3) fluorescence after 60 minutes of overexpression when compared to the empty vector control (Fig. 1d). The fluorescence value of ~4,300 arbitrary units (AU) in the empty vector control represented background fluorescence as revealed by fluorescence microscopy (Supplementary Fig. S1). DiBAC_4_(3) fluorescence was consistent with the proposed toxicity of the two DinQ variants, with DinQ-V causing higher fluorescence values than DinQ-III (~10,500 vs. ~6,700 AU, respectively). While TisB was comparable to DinQ-V, HokB caused the highest fluorescence values (~18,000 AU). We tentatively conclude that the degree of depolarization depends on the potential of the respective toxin, but cannot exclude that differences in toxin expression levels contributed to the observed differences in depolarization. Furthermore, HokB supposedly forms larger pores (~0.59 to 0.64 nm)^13^ than TisB (~0.15 nm)^17^. The larger pore size of HokB might support increased uptake of DiBAC_4_(3), which is congruent with strong depolarization of the inner membrane and ATP leakage^13^.

Formation of ROS was measured after 60 minutes of toxin overexpression using the fluorogenic dye 2′,7′-dichlorodihydrofluorescein diacetate (H_2_DCFDA). H_2_DCFDA is oxidized to the highly fluorescent 2′,7′-dichlorofluorescein (DCF) by various ROS, including hydrogen peroxide, peroxyl radicals, and peroxynitrite^24^. Importantly, H_2_DCFDA is cell-permeable and expected to enter cells irrespective of pore formation or size. All toxins, except DinQ-III, caused a significant increase in DCF fluorescence compared to the empty vector control, indicating enhanced ROS formation (Fig. 1e). TisB and DinQ-V were again comparable, causing a DCF fluorescence increase of ~2.5-fold. As expected, HokB caused the strongest increase of ~3.5-fold. The toxin-dependent increase in ROS formation, therefore, matched the degree of depolarization (compare Fig. 1d,e). The depolarizing agent carbonyl cyanide *m*-chlorophenylhydrazone (CCCP) was applied at a final concentration of 50 µM to inhibit the growth of wild type MG1655 cells (Supplementary Fig. S2). CCCP caused a significant increase in DCF fluorescence of ~3.8-fold already after 30 minutes (Fig. 1e), but did not affect DCF fluorescence in cell-free reactions (Supplementary Fig. S3). ROS measurements using fluorescein dyes can be affected by an increase in the intrinsic fluorescence of cells^28^. However, in our experiments neither toxin expression nor CCCP treatment increased the intrinsic fluorescence (fold changes of 0.85 to 0.96). Collectively, our results indicate that depolarizing toxins cause a disturbance of metabolic functions with the potential to trigger ROS formation. Whether depolarization and ROS formation causally depend on each other, or are independent outcomes of toxin expression, remains speculative (see Discussion).

### Depolarization by toxin TisB specifically induces the SoxRS regulon

**Figure 2:**
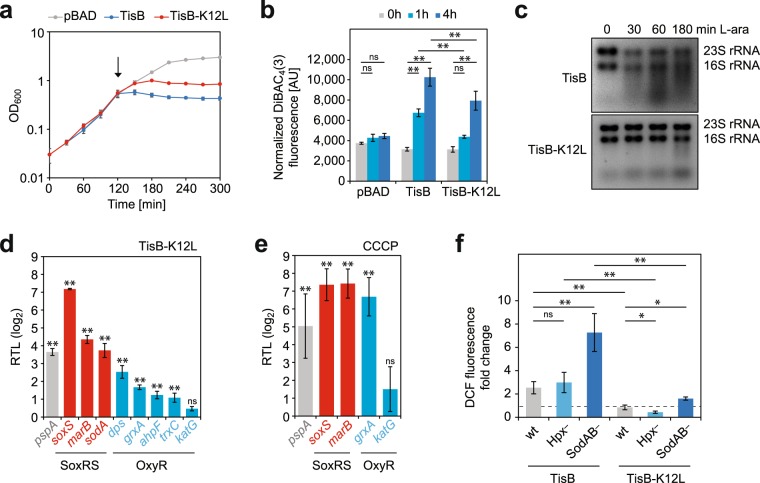
TisB expression induces the SoxRS regulon. An attenuated TisB variant was generated by amino acid exchange of lysine 12 to leucine. Ectopic expression of wild-type TisB and TisB-K12L was induced by 0.2% L-arabinose during mid-exponential growth. The empty vector (pBAD) was used as control. **(a)** Growth curves were recorded by monitoring OD_600_ in 30-min time intervals. At time point 120 min expression was induced by 0.2% L-arabinose (arrow). Data points represent the mean and error bars depict the standard deviation (n = 3). **(b)** Cellular depolarization was measured by staining with DiBAC_4_(3) at time points as indicated. Fluorescence signals were OD_600_-normalized. Data represents the mean and error bars depict the standard deviation (n = 3). For statistical analysis two-way ANOVA with post-hoc Tukey HSD was performed. Significance levels are indicated (ns: not significant, **P < 0.01). **(c)** Total RNA from toxin-overexpressing cultures was isolated at the indicated time points and analyzed on RNA quality gels. **(d**,**e)** Cultures in mid-exponential phase were either subjected to **(d)** TisB-K12L overexpression for 60 min or **(e)** CCCP treatment for 30 min. Total RNA was isolated from pre- and post-treatment cultures and analyzed by qRT-PCR to calculate log_2_ fold changes of relative transcript levels (RTL) for selected genes. Data represents the mean and error bars depict the standard deviation (n = 3). For statistical analysis two-way ANOVA with post-hoc Tukey HSD was performed. Significance levels for pre- versus post-treatment samples are indicated (ns: not significant, **P < 0.01). **(f)** ROS measurements in strains with impaired detoxification of superoxide or hydrogen peroxide. Toxins TisB and TisB-K12L were overexpressed for 60 min and H_2_DCFDA measurements performed with 0 min and 60 min samples. DCF fluorescence signals were measured in a microplate reader and OD_600_-normalized to calculate fold changes. Hpx^−^ denotes a Δ*katG* Δ*katE* Δ*ahpF* and SodAB^−^ a Δ*sodA* Δ*sodB* deletion strain. Data represents the mean and error bars depict the standard deviation (n ≥ 3). For statistical analysis two-way ANOVA with post-hoc Tukey HSD was performed. Significance levels are indicated (ns: not significant, *P < 0.05, **P < 0.01).

To further investigate toxin-dependent ROS formation, TisB was selected as an established model toxin^18,21^. TisB (29 amino acids long) forms an alpha-helix with a hydrophilic side containing five charged amino acids (Supplementary Fig. S4). In a recent screen for TisB variants with altered toxicity, we identified the positively charged amino acid lysine at position 12 to be important for toxicity (unpublished results). The exchange of lysine with leucine (K12L) generated a TisB variant with attenuated toxicity. Upon addition of L-arabinose, a wild-type strain featuring TisB-K12L had a delay in growth inhibition of ~30 minutes in comparison to non-mutated TisB (Fig. 2a), resulting in a significantly higher optical density at 300 min (*P* < 0.01, one-way ANOVA with post-hoc Tukey HSD). As expected, depolarization by TisB-K12L was delayed as well, and DiBAC_4_(3) fluorescence values did not reach the levels of non-mutated TisB after four hours of induction (~7,900 vs. ~10,300 AU, respectively; Fig. 2b). Since *tisB* mRNA levels (Fig. 1b) and protein levels (Supplementary Fig. S5) were largely unaffected by the K12L mutation, we hypothesize that the attenuated toxicity of TisB-K12L is due to impaired pore formation or less effective passage of protons across the inner membrane. Upon overexpression of non-mutated TisB, progressive degradation of 16 S and 23 S rRNAs was observed^12^, and was also confirmed here (Fig. 2c). Even though 5 S rRNA and *tisB* mRNA itself were not affected to the same extent (Supplementary Fig. S6), other transcripts might be subject to degradation in the overexpression strain, which would clearly distort their quantification. Overexpression of the TisB-K12L variant, on the other hand, did not cause obvious rRNA degradation until 180 minutes post induction (Fig. 2c). RNA samples from TisB-K12L overexpression experiments at 60 minutes post induction were compared to pre-treatment samples to assess changes in transcript levels for genes from the oxidative stress response using quantitative RT-PCR. The *pspA* gene, encoding a bifunctional protein of the envelope stress response, was chosen as a positive control, since *pspA* is known to be induced by pore-forming proteins^33^. As expected, the transcript level of *pspA* was increased ~13-fold upon overexpression of TisB-K12L (Fig. 2d). Genes from the SoxRS regulon (response to superoxide and nitric oxide) showed a similar (~13-fold for *sodA*) or even higher induction (~20-fold for *marB* and ~145-fold for *soxS*). By contrast, genes from the OxyR regulon (response to hydrogen peroxide) were only slightly affected (~6-fold for *dps*, ~3-fold for *grxA*, ~2-fold for *ahpF*, and ~2-fold for *trxC*) or not affected at all (*katG*) (Fig. 2d). Considering that *dps* is the gene with the strongest induction within the OxyR regulon upon hydrogen peroxide stress (~180-fold)^34^, the increase observed here upon TisB-K12L overexpression appears negligible. Since treatment with CCCP for 30 minutes gave the strongest increase in ROS formation (Fig. 1e), it was tested whether CCCP activates the oxidative stress response. As expected, the SoxRS regulon genes were strongly induced (~164-fold for *soxS* and ~172-fold for *marB*). Furthermore, and in contrast to TisB-K12L overexpression experiments, genes from the OxyR regulon were induced as well, as observed for *grxA* (~103-fold) (Fig. 2e). These results indicated that CCCP caused enhanced formation of both superoxide and hydrogen peroxide, while TisB-dependent depolarization failed to produce enough hydrogen peroxide to fully induce the OxyR regulon.

To further confirm our findings, TisB and TisB-K12L were overexpressed in mutants lacking ROS-detoxifying enzymes. The Hpx^−^ mutant lacks all three enzymes involved in hydrogen peroxide detoxification (Ahp, KatG, and KatE), and shows strongly enhanced DCF fluorescence upon addition of hydrogen peroxide (Supplementary Fig. S7). The SodAB^−^ mutant lacks both cytoplasmic superoxide dismutases (SodA and SodB). In the Hpx^−^ mutant and in the wild type, DCF fluorescence was increased to the same extent (2.5 to 3-fold) upon overexpression of non-mutated TisB (Fig. 2f). Surprisingly, overexpression of TisB-K12L did not increase fluorescence, neither in the wild type nor in the Hpx^−^ mutant strain (Fig. 2f). In SodAB^−^ cells, however, both non-mutated TisB and TisB-K12L provoked elevated DCF fluorescence values (Fig. 2f). These results confirmed that TisB overexpression resulted in formation of superoxide, but not hydrogen peroxide.

### TisB contributes to ROS formation upon ciprofloxacin treatment

**Figure 3:**
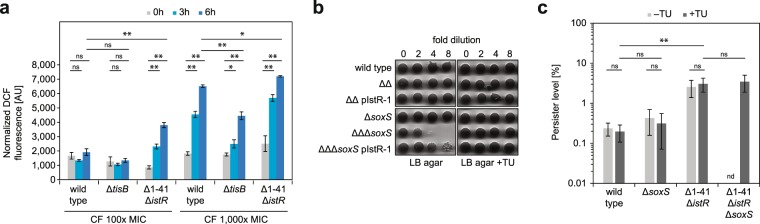
TisB-dependent ROS formation upon DNA damage and the influence of SoxS on persister formation. **(a)** ROS measurements upon antibiotic treatment. Mid-exponential cultures of wild type, Δ*tisB*, and Δ1-41 Δ*istR* were treated with ciprofloxacin at 100x MIC (1 µg mL^−1^) or 1,000x MIC (10 µg mL^−1^), and stained with H_2_DCFDA at the indicated time points. DCF fluorescence signals were measured in a microplate reader and OD_600_-normalized. Data represents the mean and error bars depict the standard deviation (n = 3). For statistical analysis three-factor ANOVA with post-hoc Tukey HSD was performed. Significance levels are indicated (ns: not significant, *P < 0.05, **P < 0.01). **(b)** Plating defect of a *soxS* deletion in strain Δ1-41 Δ*istR* (ΔΔ). Growth on solid LB medium was tested with mid-exponential cultures adjusted to approximately 10^9^ cells mL^−1^. Five µL of 1:2 dilutions were spotted on LB agar with or without 10 mM thiourea. pIstR-1 indicates constitutive expression of antitoxin IstR-1 from a plasmid. **(c)** Influence of a *soxS* deletion on wild-type and TisB-dependent persister formation. CFU counts were determined at 0 hours and 4 hours of ciprofloxacin treatment (1,000x MIC, 10 µg mL^−1^) on LB agar with or without 10 mM thiourea. Data represents the mean and error bars depict the standard deviation (n ≥ 4). For statistical analysis robust ANOVA^58^ was performed. Significance levels are indicated (ns: not significant, **P < 0.01). Persister level of strain Δ1-41 Δ*istR* Δ*soxS* was not determined (nd) on LB agar without thiourea due to the plating defect.

While plasmid-borne overexpression experiments are useful to evaluate effects of strong toxin production, chromosomal deletions are preferable to assess toxin functions under more physiological conditions. The fluoroquinolone antibiotic ciprofloxacin (CF) can be used to activate the SOS response and, consequently, TisB synthesis^18,21^. It was shown that a *tisB* deletion strain does not undergo depolarization upon CF treatment during exponential phase^18^. We therefore exposed wild-type and Δ*tisB* cultures to CF and measured DCF fluorescence over time (Fig. 3a). An increase in DCF fluorescence was only observed at very high CF concentrations (1,000x MIC). As supposed by our findings with TisB overexpression strains, the Δ*tisB* strain scored lower fluorescence values (e.g., ~4450 AU in Δ*tisB* vs. ~6500 AU in wild type after six hours of treatment). However, at lower CF concentrations (100x MIC), differences were not significant. These data indicate that, at very high ciprofloxacin concentrations, TisB contributes to ROS formation in a wild-type background. We performed the same experiment with double deletion strain Δ1-41 Δ*istR*, which lacks both the antitoxin gene *istR-1* and an inhibitory structure in the 5′ untranslated region of the *tisB* mRNA. Due to deletion of both inhibitory RNA elements, TisB production is easily excited by addition of CF, resulting in a highly persistent phenotype^18,35^. In Δ1-41 Δ*istR* cultures, DCF fluorescence increased over time and was significantly higher than in wild-type cultures irrespective of the CF concentration (Fig. 3a). Intrinsic fluorescence did not account for the changes in DCF fluorescence: strain Δ1-41 Δ*istR* did not show an increase in intrinsic fluorescence at all (fold changes of 0.93 to 0.99), and wild type and Δ*tisB* were not strongly affected (fold changes of 0.94 to 1.31). Moreover, all DCF measurements were corrected for intrinsic fluorescence. In summary, the data nicely confirmed the effects seen with plasmid-borne overexpression of TisB.

### Detoxification of superoxide is important for TisB-dependent persister formation and recovery

How does TisB-dependent formation of superoxide affect the persister life cycle of *E*. *coli*? To answer this question, we performed experiments with strain Δ1-41 Δ*istR* in comparison to wild type MG1655. Since mRNA levels of the master regulator of the superoxide response, SoxS, were strongly induced upon TisB-K12L overexpression (Fig. 2d), we tested whether the highly persistent phenotype of strain Δ1-41 Δ*istR* was affected by a *soxS* deletion. Interestingly, the Δ1-41 Δ*istR* Δ*soxS* strain exhibited a plating defect on LB agar, which was not observed in strains with only the Δ1-41 Δ*istR* or the Δ*soxS* mutations (Fig. 3b). The plating defect was largely suppressed upon antitoxin IstR-1 overexpression, and abolished when the ROS scavenger thiourea was added to the LB agar (Fig. 3b). Since addition of thiourea to LB agar plates had no effect on the outcome of persister assays (Fig. 3c), thiourea was routinely used in order to reliably determine perister levels of strain Δ1-41 Δ*istR* Δ*soxS*. The *soxS* deletion, however, had no effect on the persister level of neither wild type nor strain Δ1-41 Δ*istR* after four hours of CF treatment at 1,000x MIC (Fig. 3c). It is known that SoxS shares an overlapping regulon with the transcriptional regulators MarA and Rob^36^, and the partial redundancy of these regulators might explain why a *soxS* deletion had no effect.

**Figure 4:**
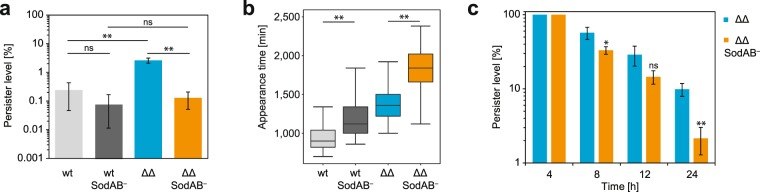
Superoxide dismutases affect TisB-dependent persister formation and recovery. Mid-exponential cultures were treated with ciprofloxacin (1,000x MIC, 10 µg mL^−1^) and plated on LB agar with 10 mM thiourea but without antibiotics to monitor CFU counts and colony growth. **(a)** CFU counts were determined at 0 hours and 4 hours to calculate persister levels in wild type (wt), strain Δ1-41 Δ*istR* (ΔΔ), and respective *sodA* and *sodB* deletions (SodAB^−^). Data represents the mean and error bars depict the standard deviation (n ≥ 7). For statistical analysis robust ANOVA^58^ was performed. Significance levels are indicated (ns: not significant, **P < 0.01). **(b)** ScanLag analysis of colony growth on LB agar after 4 hours of ciprofloxacin treatment. Appearance time indicates the first detection events of individual colonies (see Methods). Corresponding colony growth time can be found in Supplementary Figure S8. Pairwise Wilcoxon rank sum test was applied (**P < 0.01). Wild type (wt, n = 339), wt SodAB^−^ (n = 314), Δ1-41 Δ*istR* (ΔΔ, n = 1076), and ΔΔ SodAB^−^ (n = 561). **(c)** Killing kinetics of persister subpopulation. CFU counts were determined at 4, 8, 12, and 24 hours. Persister levels were calculated relative to 4-hours samples for strain Δ1-41 Δ*istR* (ΔΔ) and ΔΔ SodAB^−^. Student’s *t*-test compares ΔΔ versus ΔΔ SodAB^−^ for each time point (ns: not significant, *P < 0.05, **P < 0.01).

To further explore the role of superoxide in TisB-dependent persisters, it was tested whether directly preventing superoxide detoxification affects persistence. The SodAB^−^ mutation (Δ*sodA* and Δ*sodB*) was constructed in strain Δ1-41 Δ*istR*. Persister levels after four hours of CF treatment (1,000x MIC) were reduced >20-fold relative to the parental strain (Fig. 4a). By contrast, the SodAB^−^ mutation only caused slightly decreased (~2.7-fold) persister levels in the wild-type background (Fig. 4a). Furthermore, factor analysis (robust two-way ANOVA) revealed that the presence of *sodA* and *sodB* had a stronger contribution to persister fromation than the Δ1-41 Δ*istR* mutation. The ScanLag method^37^ was applied to monitor appearance and growth times of colonies after CF treatment (see Methods for details). If the colony growth time of a particular strain is not changed, the colony appearance time reflects the persistence time. The persistence time might be prolonged due to impaired resuscitation or recovery from the persister state^35^. The median colony appearance time was shifted from 1,360 to 1,840 minutes due to the SodAB^−^ mutation in strain Δ1-41 Δ*istR*, while in the wild-type background the same mutation only caused a shift from 900 to 1,120 minutes (Fig. 4b). Importantly, the colony growth time was largely unaffected by the SodAB^−^ mutation (Supplementary Fig. S8), demonstrating that the delayed colony appearance was due to failure in growth resumption. In summary, prevention of superoxide detoxification impaired both formation and recovery of persister cells, which was particularly evident for TisB-dependent persisters.

Persistence is typically revealed by biphasic killing kinetics upon treatment with antibiotics. While the susceptible subpopulation is rapidly killed during the first phase of the treatment, the persister subpopulation is only slowly eliminated during the second phase^38^. Killing kinetics of persisters can be affected by their wake-up kinetics, that is, how fast persisters recover and resume growth to become susceptible to antibiotics again^39^. Since persisters of strain Δ1-41 Δ*istR* SodAB^−^ showed an impaired recovery, as judged from the 8-hour shift of the median colony appearance time in comparison to strain Δ1-41 Δ*istR* (Fig. 4b), the persister subpopulation might experience less killing within the second phase of long-term killing experiments. Both strains were treated with CF (1,000x MIC) for 24 hours, revealing biphasic killing kinetics (Supplementary Fig. S9). The persister level after four hours of CF treatment was chosen as reference point (set to 100%) to calculate killing of the persister subpopulation. Contrary to initial expectations, the persister subpopulation of strain Δ1-41 Δ*istR* SodAB^−^ declined faster than observed for Δ1-41 Δ*istR* (Fig. 4c). These results indicate that killing kinetics of strain Δ1-41 Δ*istR* SodAB^−^ is not determined by wake-up kinetics, but rather by the inability to detoxify superoxide.

## Discussion

In this study, we demonstrate that small hydrophobic proteins from type I TA systems have the potential to cause elevated levels of ROS, and that the increase in ROS is consistent with the magnitude of toxin-induced depolarization (Fig. 1d,e). It appears tempting to conclude that depolarization by pore-forming toxins represents a cellular disturbance that leads to ROS formation. However, from mitochondria the exact opposite is known: depolarization of the inner membrane (i.e., lowering the membrane potential) leads to a higher flux through the electron transport chain and, consequently, declining ROS levels^40^. Even though this causal relationship is widely accepted, there are several contrary observations. For example, when the redox environment of mitochondria becomes oxidized, depolarization by protonophors (similar to CCCP) leads to elevated ROS levels, which is explained by depletion of the ROS scavenger pool^41^. The processes affecting ROS levels upon depolarization are obviously complex, and the situation in exponentially growing bacteria might also be different from mitochondria. However, a direct causal relationship between depolarization by type I toxins and ROS formation is difficult to prove without applying single-cell measurements. We can, therefore, not exclude that both observations are independent from each other. Type I toxins might depolarize the inner membrane and in parallel interfere with cellular processes to trigger ROS production. Another central question concerns whether ROS are mainly generated in dying cells. For example, membrane-disrupting antimicrobial peptides (AMPs) kill bacteria, which is partly attributable to rapid ROS formation^42,43^. In the case of AMPs, ROS are clearly linked to dying cells. In our experiments, however, this association does not necessarily hold. First of all, even though CCCP-treated cultures showed a strong increase in ROS formation (Fig. 1e), all cells survived the treatment (Supplementary Fig. S2). Secondly, upon ciprofloxacin challenge, strain Δ1-41 Δ*istR* had both higher ROS (Fig. 3a) and higher persister levels, i.e., number of surviving cells, than the wild type (Figs 3c and 4a). These data clearly indicate that it is an oversimplification to associate ROS formation with dying cells.

Our experiments indicate that superoxide is the main ROS produced upon expression of toxin TisB (Fig. 2d,f). We notice, however, that superoxide has a very low activity towards the fluorogenic dye H_2_DCFDA which was used for ROS detection. Since DCF fluorescence levels were elevated in a SodAB^−^ background (Fig. 2f), the reactive species detected by H_2_DCFDA is likely generated downstream of superoxide. Superoxide and nitric oxide cause peroxynitrite formation, for which H_2_DCFDA is highly sensitive^24^. However, we were unable to score higher DCF fluorescence values in a strain lacking the nitric oxide detoxification systems Hmp (nitric oxide dioxygenase) and NorVW (nitric oxide reductase) upon TisB overexpression (data not shown). Alternatively, increased DCF fluorescence might have originated from reaction with hydroperoxyl radicals (protonated superoxide radicals), but this remains to be tested.

It has been a long-standing debate whether ROS are produced upon antibiotic treatment^24–27^. In our hands, prolonged CF treatment triggered ROS formation only at very high CF concentrations (1,000x MIC). At lower CF concentrations (100x MIC), ROS formation was negligible, unless TisB synthesis was de-repressed (strain Δ1-41 Δ*istR*). Moreover, the Δ*tisB* strain consistently formed less ROS than the wild type, which was especially evident at 1,000x MIC (Fig. 3a). These results indicate that ROS formation depends on the antibiotic concentration, and that ROS production can be enhanced by endogenously produced factors, i.e., depolarizing type I toxins. Importantly, strain Δ1-41 Δ*istR* forms more persister cells than the wild type^18^ (Fig. 4a), suggesting that ROS production is an inevitable, but sublethal, consequence of TisB expression and probably other type I toxins. In other words, as long as detoxifying enzymes are present and active, ROS might not reach critical levels at all and persister formation is strongly favored by toxin synthesis. Interestingly, it was observed that pre-incubation of *E*. *coli* with subinhibitory concentrations of the redox-cycling drug paraquat (PQ) caused increased persister levels upon subsequent antibiotic treatment, which was attributed to upregulation of the AcrAB-TolC efflux pump as part of the SoxRS regulon^44^. However, expression of AcrAB-TolC was not essential for the positive effect exerted by PQ on persister formation^45^, and it is likely that additional members of the SoxRS regulon support persister formation. Here, we found that the SoxRS regulon member SodA and the SoxRS-independent superoxide dismutase SodB support TisB-dependent persister formation. In summary, we conclude that the TisB-dependent persister formation process itself inflicts stress on the cells due to ROS formation, and that only well-adapted cells are able to fully progress to the persister state.

What happens to cells that express high TisB levels but fail to deal with the increased ROS surge? Our data show that the inability of TisB-expressing cells to detoxify superoxide interferes with both persister formation (Fig. 4a) and recovery (Fig. 4b). It is worthwhile to carefully revisit the readout of a typical persister assay, which is the ability of a persister cell to form a colony. The failure of a single cell to form a colony can be explained by the fact that it is simply dead. Alternatively, a cell may have entered a deeper state of dormancy and does not easily resuscitate^46^. Resuscitation will either need more time or specialized conditions, as observed for VBNC cells^5^. Interestingly, VBNC cells show features of oxidative damage^47^, and the degree of oxidative damage might determine whether a cell will become a ‘shallow’ persister (wild type in Fig. 4b), ‘deep’ persister (Δ1-41 Δ*istR* SodAB^−^ in Fig. 4b), or VBNC. We recently identified alkyl hydroperoxide reductase (Ahp) as important for recovery of TisB-dependent persisters^35^, and in VBNC cells catalases play a role in resuscitation^48^. Formation of ROS and oxidative damage can therefore be expected to play crucial roles during dormancy-regrowth cycles of bacteria.

Another factor that influences colony formation is the stress provoked by the transfer of cells from liquid to solid media, a typical procedure in most persister assays. It was observed that several global stress responses, including the oxidative stress response controlled by OxyR and SoxRS, are switched on immediately after transfer^49^. While a plating defect of *oxyR* mutants is well documented in *E*. *coli* and other bacteria, an *E*. *coli soxS* deletion strain grows normally on solid media. Here, we show that de-repression of TisB synthesis in a Δ*soxS* background (strain Δ1-41 Δ*istR* Δ*soxS*) gives a synthetic plating defect (Fig. 3b). We speculate that TisB synthesis is triggered upon transfer from liquid to solid media, and that the enhanced, TisB-dependent formation of superoxide cannot be efficiently counteracted. Whether these cells decease or enter a deep state of dormancy remains an exciting question.

A recent study suggested that ROS formation triggers depolarization, which in turn favors persistence^50^. Our findings that toxin-dependent depolarization might trigger ROS formation implies a potential positive feedback loop between depolarization and ROS. Hypothetically, weakly depolarized cells with low ROS levels turn into persisters, while strongly depolarized cells accumulate high ROS levels and become VBNC or die. Parallel measurements of depolarization and ROS formation on the single-cell level might answer these questions in the future. Overall, our data support the view that decision-making with regard to persistence depends on primary (toxins) and secondary factors (stress defense systems), and that heterogeneous expression of these factors produces a continuum of dormancy^5^ within stress-tolerant subpopulations.

## Methods

### Growth conditions

*E*. *coli* strains (Supplementary Table S1) were grown under aerobic conditions in lysogeny broth (LB) at 37 °C with continuous shaking at 180 rpm. If applicable, antibiotics were added at the following concentrations: 200 µg mL^−1^ ampicillin, 50 µg mL^−1^ kanamycin, 15 µg mL^−1^ chloramphenicol and 6 µg mL^−1^ tetracycline. Over-night cultures were diluted 100-fold into fresh LB medium. For growth experiments, the optical densitiy at 600 nm (OD_600_) was adjusted to 0.05 from stationary cultures and growth was monitored using a Cell density meter model 40 (Fisher Scientific).

### Plasmid and strain construction

For HokB and DinQ overexpression plasmids, the toxin ORFs were PCR-amplified using primer pairs BA-3/BA-4 (HokB), BA-7/BA-8 (DinQ-III), and BA-7a/BA-8 (DinQ-V), respectively. An artificial Shine-Dalgarno sequence was added by forward primers BA-3, BA-7, and BA-7a (Supplementary Table S2). Vector pBAD^12^ was amplified with BA-1 and BA-2. All PCR fragments were digested with EcoRI and XbaI FastDigest^TM^ restriction enzymes (Thermo Fisher Scientific). Toxin ORFs were ligated into the pBAD backbone with T4 DNA ligase (New England Biolabs). Site-directed mutagenesis PCR was performed with primer pair K12L-for/rev using pBAD + 42 as template, followed by DpnI (Thermo Fisher Scientific) digestion. All plasmids were confirmed by sequencing (Microsynth SeqLab) and are listed in Supplementary Table S1.

Chromosomal deletion strains were constructed using the λ red genes for homologous recombination^51^. To this end, chloramphenicol acetyltransferase (*cat*) or kanamycin resistance (*kan*) genes were PCR-amplified using primers with target gene-specific overhangs of 40 bp. Corresponding DNA fragments were transformed into electrocompetent *E*. *coli* strains bearing temperature-sensitive pSIM5 plasmids for heat-inducible expression of the λ red genes^52^. Transformed strains were selected on LB agar plates supplemented with chloramphenicol (12.5 µg mL^−1^) or kanamycin (25 µg mL^−1^), respectively. Deletion of the target gene was verified by PCR using gene-specific screening primers. All primers used for cloning are listed in Supplementary Table S2. If applicable, chromosomal gene deletions were moved into recipient strains by P1 transduction. FLP-mediated flipping was performed using plasmid 709-FLPe (Gene Bridges) to generate marker-less deletion strains according to the manufacturer’s instructions.

### Measurement of physiological parameters using fluorescent dyes

For measurements with fluorescent dyes, toxin overexpression was induced at mid-exponential growth phase (OD_600_ 0.35 to 0.6) with 0.2% L-arabinose. Samples of approximately 2 × 10^8^ cells (in 500 µL) were stained with 1 µg mL^−1^ DiBAC_4_(3) (Sigma Aldrich) by incubation at room temperature for 20 min in the dark. Fluorescence was measured with excitation and emission wavelengths of 490 nm and 520 nm, respectively, using an Infinite M200 microplate reader (Tecan). Fluorescence signals were OD_600_-normalized. For H_2_DCFDA measurements, 95-µL samples (approximately 4 × 10^7^ cells) were stained with 10 µM H_2_DCFDA (Thermo Fisher Scientific) in 96-well plates by incubation in the dark at 37 °C with continuous shaking for 45 min. DCF fluorescence was measured with excitation and emission wavelengths of 492 nm and 525 nm, respectively. Fluorescence signals were background-corrected (unstained cell sample) and OD_600_-normalized.

### Persister assays and colony growth

Pre-cultures for persister assays were prepared with appropriate selection markers and supplemented with 10 mM thiourea. Over-night cultures were diluted into fresh LB medium without additives and incubated to mid-exponential growth phase (OD_600_ 0.35 to 0.6). Ciprofloxacin treatments were performed at a final concentration of 10 µg mL^−1^ (1,000x MIC). Samples were withdrawn at indicated time points and serial dilutions (in 0.9% NaCl) were plated on LB agar plates supplemented with 20 mM MgSO_4_ and with or without 10 mM thiourea. Persister levels were calculated using pre- and post-treatment samples. ScanLag^37^ analysis was performed as described previously^35^. In brief, LB agar plates from persister assays were incubated at 37 °C for at least 40 hours and scanned in 20-minutes time intervals using Epson Perfection V39 scanners. The image series was analyzed using published scripts^53^ for MatLab (MathWorks). For spot assays, approximately 10^9^ cells mL^−1^ from mid-exponential growth phase were harvested and two-fold dilution series prepared in 0.9% NaCl. Five µL of each dilution step were spotted on LB agar with or without 10 mM thiourea.

### RNA methods

Total RNA was isolated using the hot acid-phenol method as described elsewhere^54^. RNA quality was assessed on 1% agarose gels containing 1x TBE and 25 mM guanidinium thiocyanate. For Northern blot analysis, 5 µg of total RNA were separated on 8% polyacrylamide gels containing 1x TBE and 7 M urea (300 V, ~2.5 hours), followed by RNA transfer to Roti^®^-Nylon plus (Roth) membranes by semi-dry electroblotting (250 mA, 2-3 hours) and UV-crosslinking. Pre-hybridization was performed in Church buffer [0.5 M phosphate buffer (pH 7.2), 1% (w/v) bovine serum albumin, 1 mM EDTA, 7% (w/v) SDS]^55^ for one hour at 42 °C. Oligodeoxyribonucleotides (Supplementary Table S2) were 5′ end-labeled using T4 Polynucleotide Kinase (New England Biolabs) and [γ-^32^P]-ATP (Hartmann Analytic) to generate probes for detection of specific RNA species. Probes were added to the pre-hybridization mixture and hybridization was performed overnight. Membranes were washed (5x SSC, 0.01% SDS) and exposed to phosphorimaging screens (Bio-Rad). Screens were analyzed with the 1D-Quantity One software (Bio-Rad). For quantitative RT-PCR, DNA was digested using the TURBO DNA-*free*™ Kit (Invitrogen, Thermo Fisher Scientific). The Brilliant III Ultra-Fast SYBR Green QRT-PCR Master Mix (Agilent Technologies) was used for reaction mixtures, containing 1 ng µL^−1^ of total RNA. RT-PCR was performed in a C1000™ Thermal Cycler equipped with a CFX96™ Real-Time System (Bio-Rad). Cycle threshold (Ct) values were determined using the CFX Manager Software v3.1 (Bio-Rad), and relative transcript levels calculated according to the 2^−ΔΔCt^ method^56^. The *hcaT* gene was used as reference for normalization^54,57^.

### Statistical analysis

All analyses were performed with R statistical language (https://www.r-project.org/). Prior to analysis, fold changes were log_2_-transformed. In case of qRT-PCR data, ΔCt values were used for analysis. Shapiro-Wilk test was applied to assess normality of data, and Levene’s test was used to assess the equality of variances. ANOVA with post-hoc Tukey HSD was performed for multiple comparison. In case of heteroscedasticity, robust one-way and two-way ANOVA (“WRS2” package in R; functions *t2way*, *t1way*, and *lincon*; 10% trimming)^58^ was performed. For comparison of two independent groups, Student’s *t*-test was applied. ScanLag data were analyzed using pairwise Wilcoxon rank sum test. *P* values < 0.05 were considered significant.

## Supplementary information

Supplementary Information

## References

[1] Harms A, Maisonneuve E, Gerdes K (2016). Mechanisms of bacterial persistence during stress and antibiotic exposure. Science.

[2] Verstraeten N (2015). Obg and Membrane Depolarization Are Part of a Microbial Bet-Hedging Strategy that Leads to Antibiotic Tolerance. Mol. Cell.

[3] Balaban NQ, Merrin J, Chait R, Kowalik L, Leibler S (2004). Bacterial persistence as a phenotypic switch. Science.

[4] Kussell E, Leibler S (2005). Phenotypic Diversity, Population Growth, and Information in Fluctuating Environments. Science.

[5] Ayrapetyan, M., Williams, T. & Oliver, J. D. Relationship between the Viable but Nonculturable State and Antibiotic Persister Cells. *J*. *Bacteriol*. **200** (2018). 10.1128/JB.00249-18 PMC6153661 30082460

[6] Black DS, Kelly AJ, Mardis MJ, Moyed HS (1991). Structure and organization of *hip*, an operon that affects lethality due to inhibition of peptidoglycan or DNA synthesis. J. Bacteriol..

[7] Ronneau S, Helaine S (2019). Clarifying the Link between Toxin-Antitoxin Modules and Bacterial Persistence. J. Mol. Biol.

[8] Harms A, Brodersen DE, Mitarai N, Gerdes K (2018). Toxins, Targets, and Triggers: An Overview of Toxin-Antitoxin Biology. Mol. Cell.

[9] Page R, Peti W (2016). Toxin-antitoxin systems in bacterial growth arrest and persistence. Nat Chem Biol.

[10] Brantl S, Jahn N (2015). sRNAs in bacterial type I and type III toxin-antitoxin systems. FEMS Microbiol. Rev..

[11] Berghoff BA, Wagner EGH (2017). RNA-based regulation in type I toxin–antitoxin systems and its implication for bacterial persistence. Curr. Genet..

[12] Unoson C, Wagner EGH (2008). A small SOS-induced toxin is targeted against the inner membrane in *Escherichia coli*. Mol. Microbiol..

[13] Wilmaerts D (2018). The Persistence-Inducing Toxin HokB Forms Dynamic Pores That Cause ATP Leakage. MBio.

[14] Weel-Sneve R (2013). Single Transmembrane Peptide DinQ Modulates Membrane-Dependent Activities. PLoS Genet..

[15] Vogel J, Argaman L, Wagner EGH, Altuvia S (2004). The small RNA istR inhibits synthesis of an SOS-induced toxic peptide. Curr. Biol..

[16] Fernandez De Henestrosa AR (2000). Identification of additional genes belonging to the LexA regulon in *Escherichia coli*. Mol. Microbiol..

[17] Gurnev PA, Ortenberg R, Dörr T, Lewis K, Bezrukov SM (2012). Persister-promoting bacterial toxin TisB produces anion-selective pores in planar lipid bilayers. FEBS Lett..

[18] Berghoff BA, Hoekzema M, Aulbach L, Wagner EGH (2017). Two regulatory RNA elements affect TisB-dependent depolarization and persister formation. Mol. Microbiol..

[19] Shan Y (2017). ATP-Dependent Persister Formation in *Escherichia coli*. MBio.

[20] Conlon BP (2016). Persister formation in *Staphylococcus aureus* is associated with ATP depletion. Nat. Microbiol..

[21] Dörr T, Vulic M, Lewis K (2010). Ciprofloxacin causes persister formation by inducing the TisB toxin in *Escherichia coli*. PLoS Biol.

[22] Imlay JA (2013). The molecular mechanisms and physiological consequences of oxidative stress: lessons from a model bacterium. Nat. Rev. Microbiol..

[23] Imlay JA (2015). Diagnosing oxidative stress in bacteria: not as easy as you might think. Curr. Opin. Microbiol..

[24] Dwyer DJ (2014). Antibiotics induce redox-related physiological alterations as part of their lethality. Proc. Natl. Acad. Sci..

[25] Kohanski MA, Dwyer DJ, Hayete B, Lawrence CA, Collins JJ (2007). A Common Mechanism of Cellular Death Induced by Bactericidal Antibiotics. Cell.

[26] Liu Y, Imlay JA (2013). Cell Death from Antibiotics Without the Involvement of Reactive Oxygen Species. Science.

[27] Keren I (2013). Killing by bactericidal antibiotics does not depend on reactive oxygen species. Science.

[28] Paulander W (2014). Bactericidal Antibiotics Increase Hydroxyphenyl Fluorescein Signal by Altering Cell Morphology. PLoS One.

[29] Brynildsen MP, Winkler JA, Spina CS, MacDonald IC, Collins JJ (2013). Potentiating antibacterial activity by predictably enhancing endogenous microbial ROS production. Nat. Biotechnol..

[30] Fozo EM (2008). Repression of small toxic protein synthesis by the Sib and OhsC small RNAs. Mol. Microbiol..

[31] Gu M, Imlay JA (2011). The SoxRS response of *Escherichia coli* is directly activated by redox-cycling drugs rather than by superoxide. Mol. Microbiol..

[32] Liochev SI, Benov L, Touati D, Fridovich I (1999). Induction of the *soxRS* Regulon of *Escherichia coli* by Superoxide. J. Biol. Chem..

[33] Manganelli R, Gennaro ML (2017). Protecting from Envelope Stress: Variations on the Phage-Shock-Protein Theme. Trends Microbiol..

[34] Zheng M (2001). DNA Microarray-Mediated Transcriptional Profiling of the *Escherichia coli* Response to Hydrogen Peroxide. J. Bacteriol..

[35] Spanka D-T, Konzer A, Edelmann D, Berghoff BA (2019). High-Throughput Proteomics Identifies Proteins With Importance to Postantibiotic Recovery in Depolarized Persister Cells. Front. Microbiol..

[36] Martin RG, Rosner JL (2002). Genomics of the *marA/soxS/rob* regulon of *Escherichia coli*: identification of directly activated promoters by application of molecular genetics and informatics to microarray data. Mol. Microbiol..

[37] Levin-Reisman I (2010). Automated imaging with ScanLag reveals previously undetectable bacterial growth phenotypes. Nat. Methods.

[38] Brauner A, Fridman O, Gefen O, Balaban NQ (2016). Distinguishing between resistance, tolerance and persistence to antibiotic treatment. Nature Reviews Microbiology.

[39] Jõers A, Kaldalu N, Tenson T (2010). The frequency of persisters in *Escherichia coli* reflects the kinetics of awakening from dormancy. J. Bacteriol..

[40] Berry BJ, Trewin AJ, Amitrano AM, Kim M, Wojtovich AP (2018). Use the Protonmotive Force: Mitochondrial Uncoupling and Reactive Oxygen Species. J. Mol. Biol..

[41] Aon MA, Cortassa S, O’Rourke B, Redox-optimized ROS (2010). balance: a unifying hypothesis. Biochim. Biophys. Acta.

[42] Choi H, Yang Z, Weisshaar JC (2017). Oxidative stress induced in *E*. *coli* by the human antimicrobial peptide LL-37. PLOS Pathog..

[43] Choi H, Yang Z, Weisshaar JC (2015). Single-cell, real-time detection of oxidative stress induced in *Escherichia coli* by the antimicrobial peptide CM15. Proc. Natl. Acad. Sci. USA.

[44] Wu Y, Vulić M, Keren I, Lewis K (2012). Role of oxidative stress in persister tolerance. Antimicrob. Agents Chemother..

[45] Mosel M, Li L, Drlica K, Zhao X (2013). Superoxide-Mediated Protection of *Escherichia coli* from Antimicrobials. Antimicrob. Agents Chemother..

[46] Pu Y (2019). ATP-Dependent Dynamic Protein Aggregation Regulates Bacterial Dormancy Depth Critical for Antibiotic Tolerance. Mol. Cell.

[47] Desnues B (2003). Differential oxidative damage and expression of stress defence regulons in culturable and non-culturable *Escherichia coli* cells. EMBO Rep..

[48] Martins PMM, Merfa MV, Takita MA, De Souza AA (2018). Persistence in Phytopathogenic Bacteria: Do We Know Enough? *Front*. Microbiol..

[49] Cuny C, Lesbats M, Dukan S (2007). Induction of a global stress response during the first step of *Escherichia coli* plate growth. Appl. Environ. Microbiol..

[50] Wang T, El Meouche I, Dunlop MJ (2017). Bacterial persistence induced by salicylate via reactive oxygen species. Sci. Rep..

[51] Datsenko KA, Wanner BL (2000). One-step inactivation of chromosomal genes in *Escherichia coli* K-12 using PCR products. Proc. Natl. Acad. Sci. USA.

[52] Datta S, Costantino N, Court DL (2006). A set of recombineering plasmids for gram-negative bacteria. Gene.

[53] Levin-Reisman, I., Fridman, O. & Balaban, N. Q. ScanLag: High-throughput Quantification of Colony Growth and Lag Time. *J*. *Vis*. *Exp*. e51456 (2014). 10.3791/51456 PMC4215631 25077667

[54] Berghoff BA, Karlsson T, Källman T, Wagner EGH, Grabherr MG (2017). RNA-sequence data normalization through in silico prediction of reference genes: the bacterial response to DNA damage as case study. BioData Min..

[55] Church GM, Gilbert W (1984). Genomic sequencing. Proc. Natl. Acad. Sci..

[56] Livak KJ, Schmittgen TD (2001). Analysis of relative gene expression data using real-time quantitative PCR and the 2(-Delta Delta C(T)) Method. Methods.

[57] Zhou K (2011). Novel reference genes for quantifying transcriptional responses of *Escherichia coli* to protein overexpression by quantitative PCR. BMC Mol. Biol..

[58] Mair, P. & Wilcox, R. Robust statistical methods in R using the WRS2 package. *Behav*. *Res*. *Methods*, 10.3758/s13428-019-01246-w (2019). 10.3758/s13428-019-01246-w 31152384

[59] Omasits U, Ahrens CH, Müller S, Wollscheid B (2014). Protter: interactive protein feature visualization and integration with experimental proteomic data. Bioinformatics.

[60] Kahsay RY, Gao G, Liao L (2005). An improved hidden Markov model for transmembrane protein detection and topology prediction and its applications to complete genomes. Bioinformatics.

