# Supplementary material for: Type I toxin-dependent generation of superoxide affects the persister life cycle of Escherichia coli
Source: Sci Rep. 2019 Oct 3;9:14256. doi: 10.1038/s41598-019-50668-1 (PMC6776643; doi:10.1038/s41598-019-50668-1)
Supplement: Supplementary file 1 — Supplementary Information [file 41598_2019_50668_MOESM1_ESM.pdf]

Supplementary Information

Type I toxin-dependent generation of superoxide affects the persister life cycle of *Escherichia coli*

Daniel Edelmann and Bork A. Berghoff

Institute for Microbiology and Molecular Biology, Justus Liebig University Giessen, Giessen, Germany

This file contains:

Supplementary Figures S1-S9

Supplementary Tables S1-S2

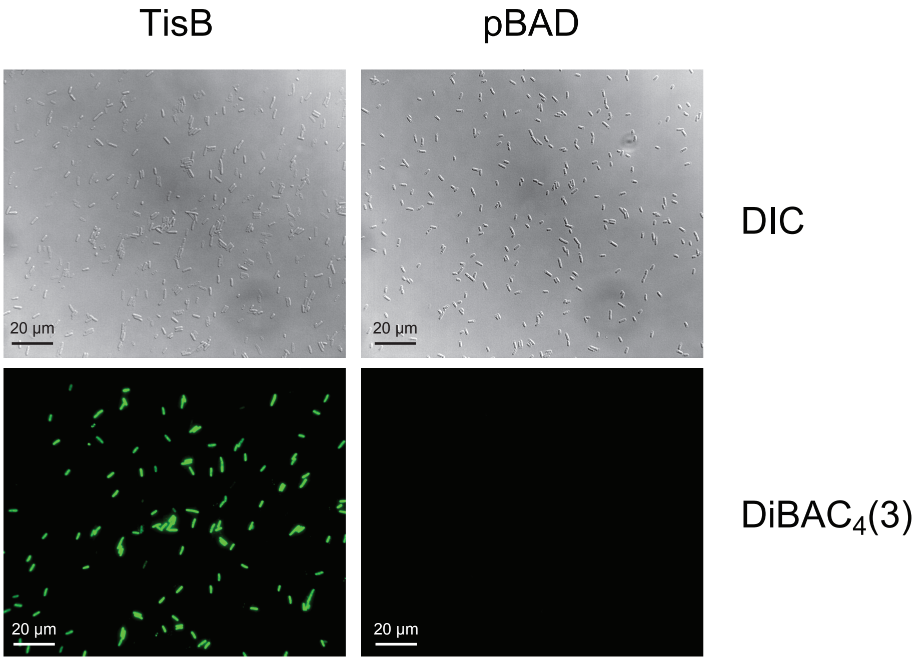

Supplementary Figure S1. Fluorescence microscopy of DiBAC$_4$(3)-stained cells.
E. coli wild type MG1655 harboring a TisB overexpression plasmid or an empty vector (pBAD) was treated with L-arabinose (final concentration 0.2%) during exponential phase for one hour. Samples were stained with DiBAC$_4$(3) and analyzed by fluorescence microscopy. Fluorescence images were recorded with a Leica DMI 6000 B inverse microscope (Leica) using a custom filter set (T495lpxr, ET525/50m; Chroma Technology). Images were processed with the ImageJ-based Fiji tool. DIC: differential interference contrast.

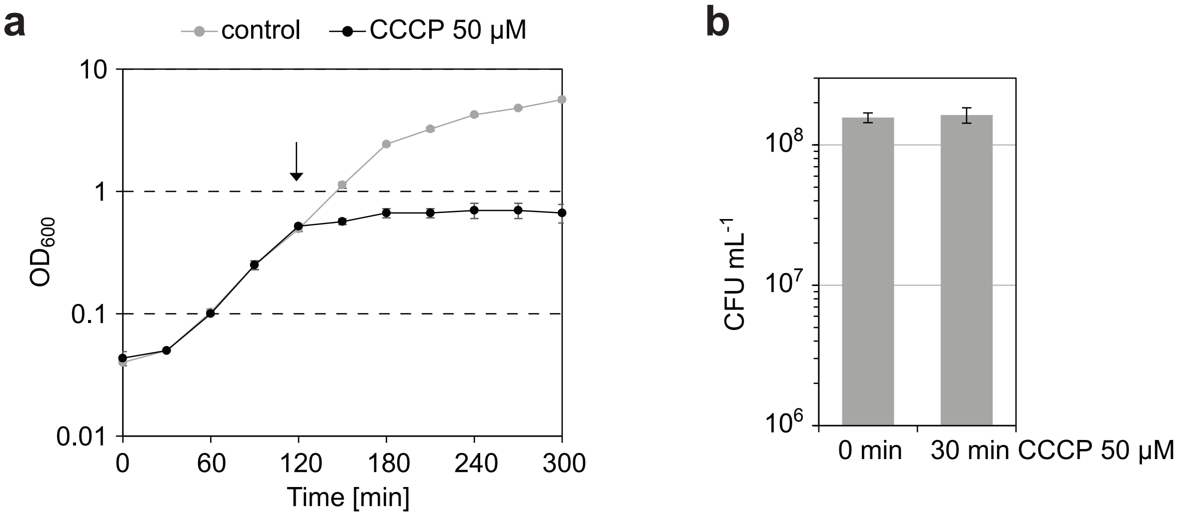

Supplementary Figure S2. Growth inhibition by CCCP.
(a) *E. coli* wild type MG1655 was treated with the depolarizing agent CCCP at a final concentration of 50 µM during exponential phase (indicated by an arrow; black curve). A control culture was left untreated (grey curve). Growth was monitored by OD_{600} measurements. Results represent the mean of biological triplicates and error bars depict the standard deviation. OD_{600} values differ significantly between control and CCCP treatment for time points later than 120 min ($p < 0.01$, Student's *t*-test).
(b) CFU counts per mL of *E. coli* wild type MG1655 after 30 min of CCCP treatment (50 µM). Results represent the mean of four biological replicates and error bars depict the standard deviation. No significant difference was detected by Student's *t*-test.

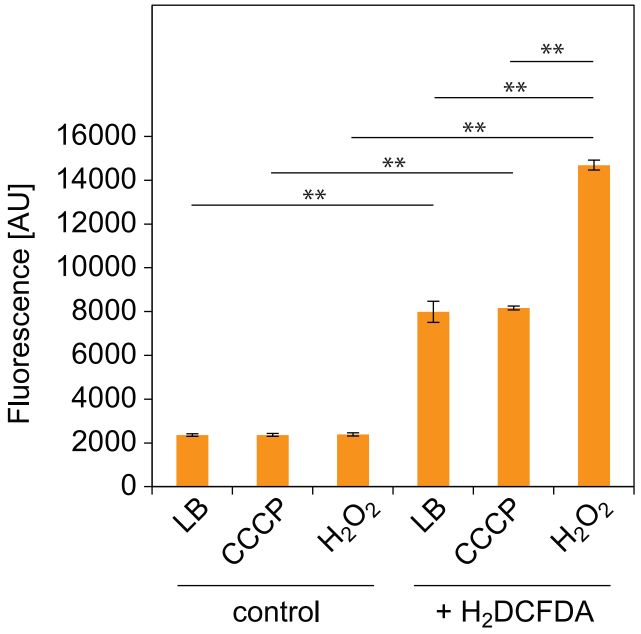

Supplementary Figure S3. DCF fluorescence upon addition of chemicals in cell-free reactions. Pure LB medium, LB medium supplemented with 50 µM CCCP, and LB medium with 10 mM hydrogen peroxide (H_2O_2) were incubated for 30 min at 37°C. Afterwards, 95-µl samples were transferred to a 96-well plate and either incubated with the fluorogenic dye H_2DCFDA (final concentration 10 µM) or without H_2DCFDA (control) for 45 min at 37°C in the dark. Fluorescence was monitored with an Infinite M200 microplate reader (Tecan) using excitation and emission wavelength of 492 nm and 525 nm, respectively. Results represent the mean and error bars depict the standard deviation (n = 3). For statistical analysis two-way ANOVA with post-hoc Tukey HSD was performed. Significance levels are indicated (** $P < 0.01$).

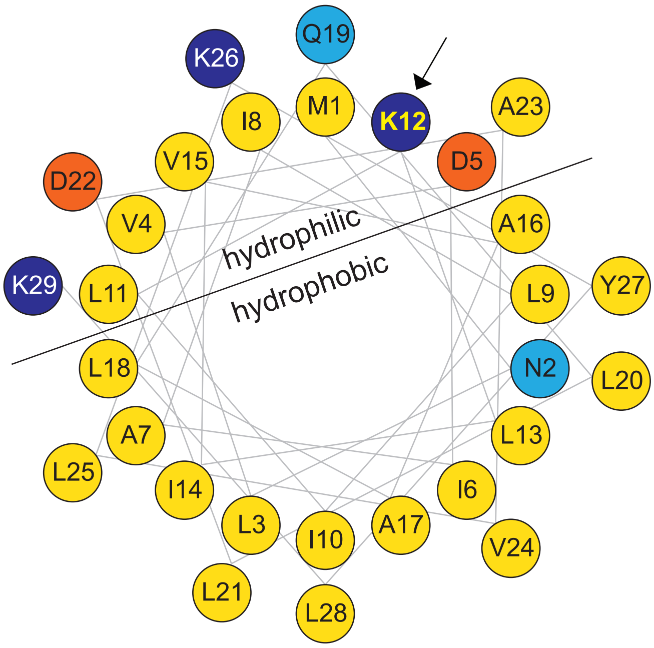

Supplementary Figure S4. Helical wheel projection of toxin TisB.
Positions of the 29 amino acids within the α-helix are shown. The α-helix has a hydrophilic and hydrophobic face. Hydrophobic side chains (yellow); polar residues (light blue); positively charged residues (dark blue); negatively charged residues (red). The lysine at position 12 (indicated by an arrow) was exchanged with a leucine in the TisB-K12L variant.

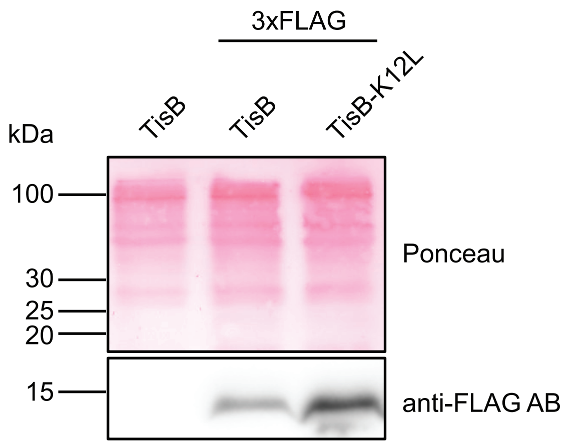

Supplementary Figure S5. Western blot analysis of 3xFLAG-TisB.
TisB variants were expressed in *E. coli* MG1655 from pBAD plasmids using L-arabinose (0.2%) for 15 minutes. Cells were lysed on ice by sonication in phosphate buffer (50 mM, pH 7.2). After removal of intact cells by centrifugation (600 rpm), membrane fractions were collected by ultracentrifuagtion (85.000 g, 45 min, 4°C). Membrane pellets were dissolved in phosphate buffer containing 0.2% sodium lauroyl sarcosinate. After precipitation of proteins with acetone, proteins were resolved in phosphate buffer, mixed with SDS sample buffer, and 50 µg were loaded onto tricine-SDS polyacrylamide-gels (16%). Proteins were tranferred to PVDF membranes by semi-dry electroblotting. Membranes were stained with Ponceau (loading control), and afterwards incubated with an anti-FLAG antibody for detection of 3xFLAG-TisB and 3xFLAG-TisB-K12L. TisB without 3xFLAG-tag served as negative control.

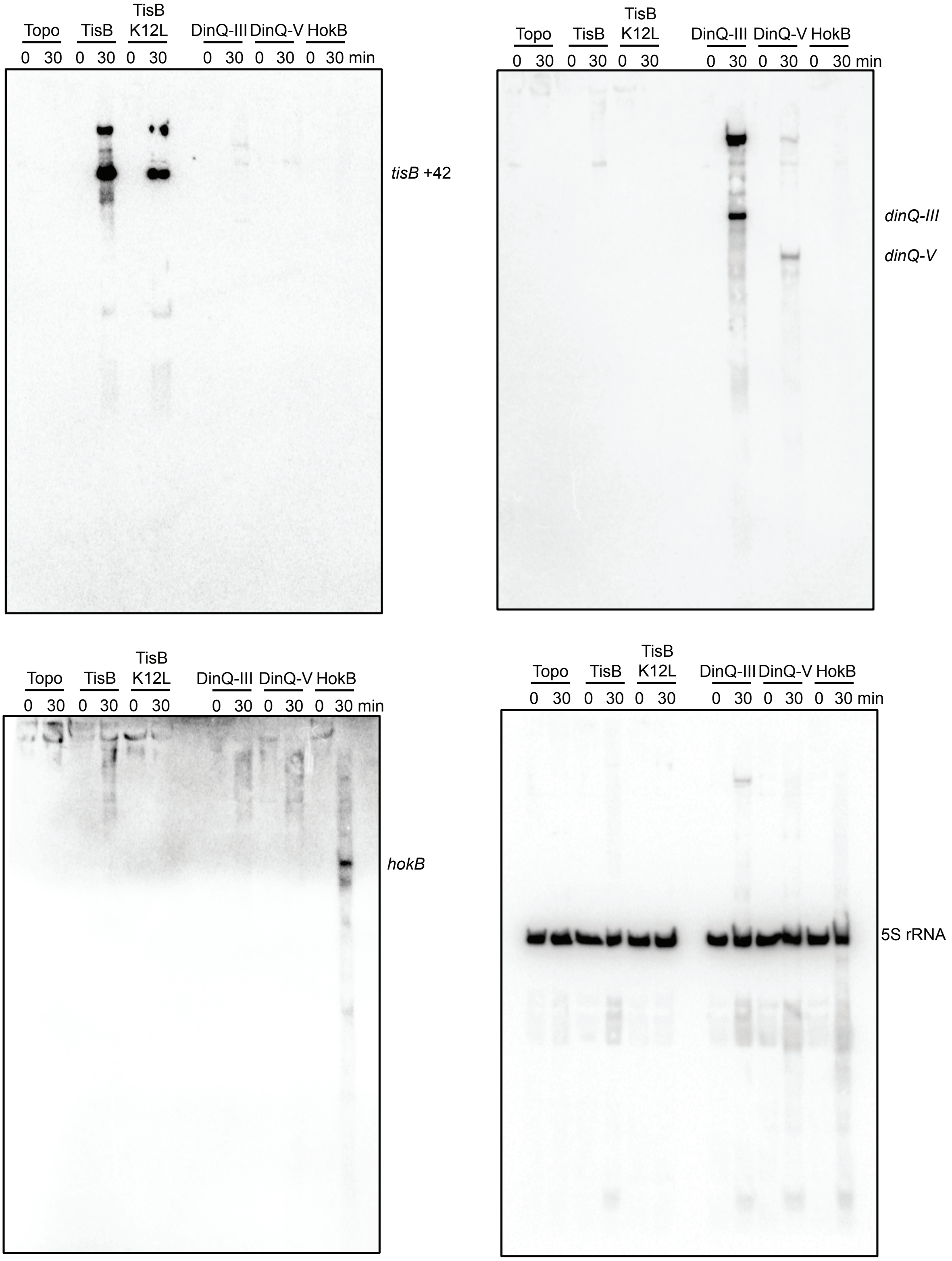

Supplementary Figure S6. Northern blot analysis of toxin mRNAs.
Total RNA was isolated at 0 min and 30 min post induction with L-arabinose (0.2%). Analysis with toxin-specific probes confirmed specific toxin overexpression. An empty vector (pBAD) was used as negative control. 5S rRNA was probed as loading control. Shown are the full scans which were used to compile Figure 1b in the main text.

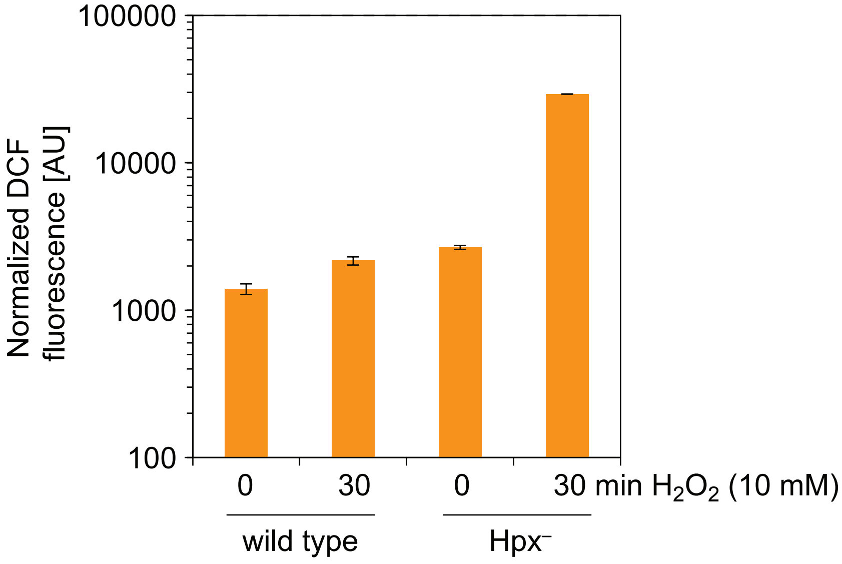

Supplementary Figure S7. *In vivo* DCF fluorescence upon addition of hydrogen peroxide.
E. coli wild type MG1655 and the Hpx⁻ mutant were grown in LB medium and treated with hydrogen peroxide (H_2O_2) at a final concentration of 10 mM during exponential phase. Samples before and 30 min after H_2O_2 treatment were used for ROS measurements. For this purpose, 95 µl cell suspension were transferred to a 96-well plate and either incubated with the fluorogenic dye H_2DCFDA (final concentration 10 µM) or without H_2DCFDA (background control) for 45 min at 37°C in the dark. Fluorescence was monitored with an Infinite M200 microplate reader (Tecan) using excitation and emission wavelength of 492 nm and 525 nm, respectively. Values were background-corrected and normalized to OD_{600}. Results represent the mean of biological duplicates with technical triplicates each. Error bars depict the standard deviation.

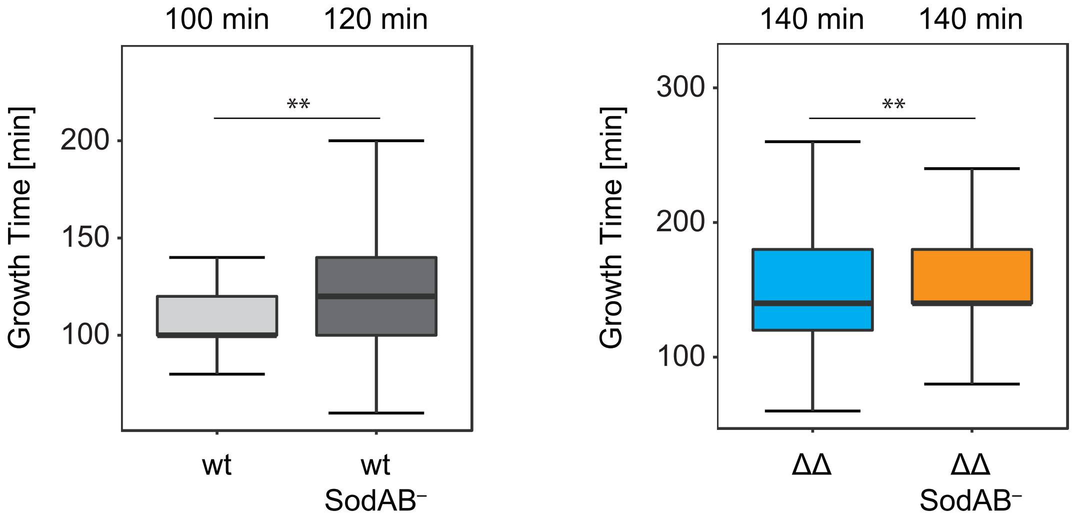

Supplementary Figure S8. Boxplots of colony growth times from ScanLag experiments.
Colony growth after ciprofloxacin treatment (1,000x MIC) was monitored over time using regular scanners, and images were analyzed according to published protocols in MATLAB (MathWorks). *E. coli* wild type MG1655 (wt; n=339), a *sodA* and *sodB* deletion strain (wt SodAB$^-$; n=314), strain Δ1-41 Δ*istR* (ΔΔ; n=1076), and a *sodA* and *sodB* deletion in strain Δ1-41 Δ*istR* (ΔΔSodAB$^-$; n=561) were analyzed. Median growth times are indicated. Pairwise Wilcoxon rank sum test was applied (** $P < 0.01$).

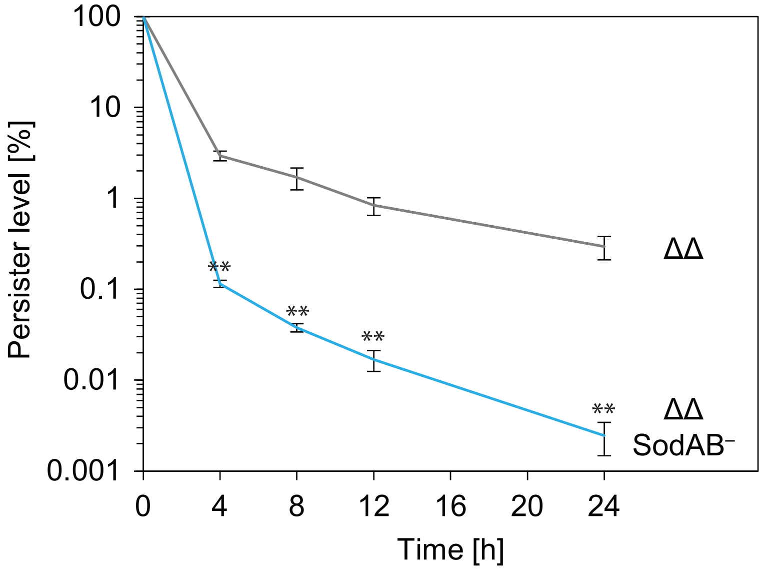

Supplementary Figure S9. Killing curve experiment with ciprofloxacin.
E. coli strain Δ1-41 Δ*istR* (ΔΔ) and a *sodA* and *sodB* deletion in strain Δ1-41 Δ*istR* (ΔΔSodAB^{-}) were treated with ciprofloxacin (1,000x MIC) during exponential phase, and persister levels were calculated over time by means of CFU counts. Results represent the mean and error bars depict the standard deviation (n = 3). Student's *t*-test compares ΔΔ versus ΔΔ SodAB^{-} for each time point (** $P < 0.01$).

Supplementary Table 1. Strains and plasmids used in this study.

Strain	Relevant features	Source/Reference
MG1655	K-12 F^- λ^-	Lab strain
Δ*tisAB*::FRT	Markerless deletion of *tisAB*	Dörr *et al.*, 2010
B133	Scarless deletion of +1-41 region of *tisB* 5'-UTR and deletion of *istR* (Δ1-41 Δ*istR::frt-kan-frt*)	Berghoff *et al.*, 2017
GC-59	Elimination of *kan* in B133 by FLP-mediated recombination (Δ1-41 Δ*istR*::FRT)	Spanka *et al.*, 2019
GC-38	Deletion of *soxS* with *cat* (Δ*soxS::cat*) in MG1655	This study
DE-15	Deletion of *soxS* with *cat* (Δ*soxS::cat*) in B133	This study
DE-71	Deletion of *sodA* with *kan* (Δ*sodA::kan*) in MG1655	This study
CB-12	Deletion of *sodB* with *cat* (Δ*sodB::cat*) in MG1655	This study
DE-79	Δ*sodB::cat* Δ*sodA::kan* in MG1655 (P1 from DE-71 to CB-12)	This study
DE-76	Δ1-41 Δ*istR*::FRT Δ*sodB::cat* (P1 from CB-12 to GC-59)	This study
DE-81	Δ1-41 Δ*istR*::FRT Δ*sodB::cat* Δ*sodA::kan* (P1 from DE-71 to DE-76)	This study
DTS-5	Deletion of *ahpF* with *cat* (Δ*ahpF::cat*) in MG1655	Spanka *et al.*, 2019
GC-54	Deletion of *katG* with *cat* (Δ*katG::cat*) in MG1655	This study
GC-56	Deletion of *katE* with *kan* (Δ*katE::kan*) in GC-54	This study
GC-86	Elimination of *kan* and *cat* in GC-56 by FLP-mediated recombination (MG1655 Δ*katG*::FRT Δ*katE*::FRT)	This study
GC-88	MG1655 Δ*katG*::FRT Δ*katE*::FRT Δ*ahpF::cat* (P1 from DTS-5 to GC-86); Hpx^-	This study
**Plasmid**	**Relevant features**	**Source/Reference**
pSIM5	λ red expression vector, pSC101 *ori*, $repA^{ts}$, Tet^R	Datta *et al.*, 2006
709-FLPe	FLPe expression plasmid, pSC101-ts *ori*, Amp^R	Gene Bridges
pBAD	modified pBAD-TOPO, Amp^R	Unoson and Wagner, 2008
pBAD+42	pBAD with *tisB* from +42 to +354	Unoson and Wagner, 2008
pBAD+42 -3xFLAG	pBAD with *tisB* from +42 to +354, 3xFLAG tag inserted 3 nt downstream of *tisB* AUG	Unoson and Wagner, 2008
pBAD+42-K12L	pBAD+42 with AAA to CTG mutation at codon 12 (K12L)	This study
pBAD+42-K12L -3xFLAG	pBAD+42-3xFLAG with AAA to CTG mutation at codon 12 (K12L)	This study
pBAD-*dinQ-III*	pBAD with *dinQ-III* ORF and artificial SD sequence	This study
pBAD-*dinQ-V*	pBAD with *dinQ-V* ORF and artificial SD sequence	This study
pBAD-*hokB*	pBAD with *hokB* ORF and artificial SD sequence	This study
pJV3H22	IstR-1 transcribed from P_L promoter in pZE12-luc, ColE1 *ori*, Amp^R	Vogel *et al.*, 2004

References

Berghoff, B.A., Hoekzema, M., Aulbach, L., and Wagner, E.G.H. (2017) Mol Microbiol 103: 1020–1033.
Datta, S., Costantino, N., and Court, D.L. (2006) Gene 379: 109–115.
Dörr T, Vulić M, Lewis K. (2010) PLoS Biol 8: e1000317.
Spanka, D.-T., Konzer, A., Edelmann, D., and Berghoff, B.A. (2019) Front Microbiol 10: 378.
Unoson, C., and Wagner, E.G.H. (2008) Mol Microbiol 70: 258–270.
Vogel, J., Argaman, L., Wagner, E.G.H., and Altuvia, S. (2004) Curr Biol 14: 2271–2276.

Supplementary Table 2. Oligodeoxyribonucleotides used in this study.

Name	Sequence (5' to 3')	Description
soxS-KO-1	AGATGAATTAACGAACTGAACACTGAAAAGAGGCAGA TTTGCTCATATGAATATCCTCCTTAG	chr. deletion of *soxS* with *cat*
soxS-KO-2	AGCAATTACCCGCGCGGGAGTTAACGCGCGGGCAATA AAAGCCTTTGAGTGAGCTGATAC	chr. deletion of *soxS* with *cat*
soxS-KO-scr1	GTTAGCAGCGCTTTAATGCG	screening of chr. *soxS* deletion
soxS-KO-scr2	CGCTATTGCCAGGGATG	screening of chr. *soxS* deletion
katG-KO-1	TGTGTATCGTAACGGTAACACTGTAGAGGGGAGCA CATTGGCTCATATGAATATCCTCCTTAG	chr. deletion of *katG* with *cat*
katG-KO-2	TTCAGCGACTGCCAGCAAGCAGCCGCTGAACGG GGTCAGAGCCTTTGAGTGAGCTGATAC	chr. deletion of *katG* with *cat*
katG-scr-1	CTCAACTATCGCATCCG	screening of chr. *katG* deletion
katG-scr-2	CGCCCACTATACGCTG	screening of chr. *katG* deletion
katE-KO-1	ACAGCGGCCCTTTCAGTAATAAATTAAGGAGAC GAGTTCATGTAGGCTGGAGCTGCTTC	chr. deletion of katE with *kan*
katE-KO-2	ATGTAAATCATTTGAGGCGGCGCAATTGCGCCG CCTCCCAGAGTGAGCTGATACCGCTCG	chr. deletion of katE with *kan*
katE-scr-1	GGATCTGGCTGGTGGTC	screening of chr. *katE* deletion
katE-scr-2	CGGATAAGGCGTTCACG	screening of chr. *katE* deletion
sodB-KO-1	CTATTGTACGTATGCAAATTAATAATAAAGGAG AGTAGCAGCTCATATGAATATCCTCCTTAG	chr. deletion of *sodB* with *cat*
sodB-KO-2	AGCGTAGCGCTTCAGGCAATGCTGCATTTGCCA TCAGTTAGCCTTTGAGTGAGCTGATAC	chr. deletion of *sodB* with *cat*
sodB-scr-1	CCCTGTTGTTACGGCAACAG	screening of chr. *sodB* deletion
sodB-scr-2	GCGTAGGCCTGATAAGCG	screening of chr. *sodB* deletion
sodA-KO-1	GGCATTAACAATCGGCCGCCCGACAATACTGGA GATGAATTGTAGGCTGGAGCTGCTTC	chr. deletion of *sodA* with *kan*
sodA-KO-2	TATGCGGCCTATACGCCTCATTGCAGCAGGCGG CAAATGAGAGTGAGCTGATACCGCTCG	chr. deletion of *sodA* with *kan*
sodA-scr-1	TCACATCTCGGGCATTTTCC	screening of chr. *sodA* deletion
sodA-scr-2	GGG CTG ATG CGT AAT TCC TC	screening of chr. *sodA* deletion
K12L-for	TTATCCTCCTGCTCATTGTTGCAG	AAA to CTG exchange at codon 12 in *tisB* (K12L)
K12L-rev	CAATGAGCAGGAGGATAAGAATGGC	AAA to CTG exchange at codon 12 in *tisB* (K12L)
BA-1	CC**GAATTC**GAGAAACAGTAGAGAGTTG	pBAD amplification, **EcoRI**
BA-2	CC**TCTAGA**TGTTTTGGCGGATGAGAGA	pBAD amplification, **XbaI**
BA-3	CC**GAATTC**AGAGAAAGAGGAGAAATACTAGAT GAAGCACAACCCTCTGG	*hokB* amplification, **EcoRI**, artificial UTR/SD
BA-4	CC**TCTAGA**GTGTAAAAGGGGTGCCATG	*hokB* amplification, **XbaI**
BA-7	CC**GAATTC**AGAGAAAGAGGAGAAATACTAGAT GAGTAAGCGGATGCATTC	*dinQ-III* amplification, **EcoRI**, artificial UTR/SD

continued

BA-7a	CC**GAATTC**AGAGAAAGAGGAGAAATACTAGGT GATTGATAAAGCAATCATCG	*dinQ-V* amplification, **EcoRI**, artificial UTR/SD
BA-8	CC**TCTAGA**GTTAAAGGGCTAAGAGTAGTGTG	*dinQ* amplification, **XbaI**
hcaT-1	ATCCGTCCGACGATTCAG	qRT-PCR primer for *hcaT*
hcaT-2	CCGTAATAGGCCGCATGT	qRT-PCR primer for *hcaT*
pspA-1	TGGTGGACGATACGCTGG	qRT-PCR primer for *pspA*
pspA-2	TGCCGCCTGATGACGTAA	qRT-PCR primer for *pspA*
soxS-1	CGAATGTTCCGCACGGTGAC	qRT-PCR primer for *soxS*
soxS-2	CAACTCAACGGCGGCCAGTA	qRT-PCR primer for *soxS*
marB-1	GCTTATTCTCTTTTCCGCGC	qRT-PCR primer for *marB*
marB-2	GCATCCGACTTATCACTGCC	qRT-PCR primer for *marB*
sodA-1	TGGGCCTGGATGTGTGGGA	qRT-PCR primer for *sodA*
sodA-2	CCGCTGCTTCGTCCCAGT	qRT-PCR primer for *sodA*
dps-1	CCGCAACGATGTCTCCGA	qRT-PCR primer for *dps*
dps-2	GCATGTTCCAGTGCGCTT	qRT-PCR primer for *dps*
grxA-1	TTGAGCAATGAACGCGATGA	qRT-PCR primer for *grxA*
grxA-2	CCGCCGATATGTTGCTGATC	qRT-PCR primer for *grxA*
ahpF-1	AACGTGCGGCAGAAGAGC	qRT-PCR primer for *ahpF*
ahpF-2	TTCGCCCATCAGACCGGT	qRT-PCR primer for *ahpF*
trxC-1	TCAGGCCATCAATCGCATTCCC	qRT-PCR primer for *trxC*
trxC-2	TCACCGGTCGCATTAATCACCT	qRT-PCR primer for trxC
katG-1	CCCGTGCCTGGTTCAAAC	qRT-PCR primer for *katG*
katG-2	TGCTCGGTCGGGTTGTAG	qRT-PCR primer for *katG*
TisB-probe	TTACTTCAGGTATTTCAGAACAGCATCAAGCAGTTGCA GTGCTGCAACAATGAGTTTGAGGATAAGAATGGCGAT ATCCACCAGGTTCAT	Northern blotting probe, end-labeling for detection of *tisB* mRNA
DinQ-probe	TCAGTTCAGAAGCTGAAGCAGAAAGCGGATCAGTTCC AGCAGCGCAATTAACGCCCCTAGAACGATGATTGCTTT ATCAATCAC	Northern blotting probe, end-labeling for detection of *dinQ* mRNA
HokB-probe	TTACCTGGACGTGCAGGCCATGAGCGCAGCAACCTCCT TATCACCGTCCCGGAACCGCAGTTCGTAGAGCGTTTGT CGGGTC	Northern blotting probe, end-labeling for detection of *hokB* mRNA
5S-probe-2	CCTGGCAGTTCCCTACTCTCGCATGAGGAG	Northern blotting probe, end-labeling for detection of 5S rRNA
